# High-Resolution Microendoscopy for the Detection of Cervical Neoplasia in Low-Resource Settings

**DOI:** 10.1371/journal.pone.0044924

**Published:** 2012-09-18

**Authors:** Mary K. Quinn, Tefo C. Bubi, Mark C. Pierce, Mukendi K. Kayembe, Doreen Ramogola-Masire, Rebecca Richards-Kortum

**Affiliations:** 1 Department of Bioengineering, Rice University, Houston, Texas, United States of America; 2 Department of Biomedical Engineering, Rutgers, The State University of New Jersey, Piscataway, New Jersey, United States of America; 3 National Health Laboratory-Princess Marina Hospital, Gaborone, Botswana; 4 School of Medicine, University of Botswana, Gaborone, Botswana; The Chinese University of Hong Kong, Hong Kong

## Abstract

Cervical cancer is the second leading cause of cancer death among women in developing countries. Developing countries often lack infrastructure, cytotechnologists, and pathologists necessary to implement current screening tools. Due to their low cost and ease of interpretation at the point-of-care, optical imaging technologies may serve as an appropriate solution for cervical cancer screening in low resource settings. We have developed a high-resolution optical imaging system, the High Resolution Microendoscope (HRME), which can be used to interrogate clinically suspicious areas with subcellular spatial resolution, revealing changes in nuclear to cytoplasmic area ratio. In this pilot study carried out at the women's clinic of Princess Marina Hospital in Botswana, 52 unique sites were imaged in 26 patients, and the results were compared to histopathology as a reference standard. Quantitative high resolution imaging achieved a sensitivity and specificity of 86% and 87%, respectively, in differentiating neoplastic (≥CIN 2) tissue from non-neoplastic tissue. These results suggest the potential promise of HRME to assist in the detection of cervical neoplasia in low-resource settings.

## Introduction

Cervical cancer is the third most common type of cancer among women worldwide. In 2008, there were an estimated 530,000 new cases and more than 275,000 deaths due to cervical cancer. Over 85% of new cases of cervical cancer and deaths due to cervical cancer occur in developing countries where screening programs for early detection are either inadequate or unavailable [1].

In the United States and many other high-resource countries, organized screening programs based on the Pap (Papanicolaou) smear and human papillomavirus (HPV) tests enable early diagnosis and treatment of precancerous lesions. An abnormal Pap smear test or HPV test is followed by colposcopy. If abnormalities are evident under colposcopy, a biopsy is required to confirm disease before treatment. The widespread use of Pap smears, colposcopy, biopsy, and treatment of pre-cancerous lesions has led to dramatic reductions in the incidence and mortality of cervical cancer in the United States [2]. However, this process requires multiple patient visits and highly trained personnel to read cytology and biopsy results. In resource-constrained settings, the increased risk of patient loss to follow-up and lack of trained personnel and laboratories often make this screening paradigm ineffective.

HPV testing has been explored in developing countries and the available data have demonstrated the potential of HPV testing to reduce both the mortality and long term risk of cervical cancer [3]. However, currently available HPV tests require six hours to produce a result, necessitating multiple visits for screening and treatment. More recently, rapid HPV tests have emerged which may offer a potential solution, but are yet to become commercially available [4].

Alternative early cervical cancer detection methods that are accurate, low cost, and that allow immediate diagnosis and treatment in a single visit are crucial for low-resource countries [5]. Visual inspection with acetic acid (VIA) is the most widely available screening modality in developing countries. This is a simple visual test, in which the cervix is examined with the naked eye before and after application of 3 to 5% acetic acid (vinegar). Upon application of the acetic acid, suspicious areas turn white. VIA has been explored in various developing countries as an alternative to cytology, and has shown promising results in detecting high-grade dysplasia or invasive carcinomas [6], [7]. Although not as extensively used as VIA, visual inspection with Lugol's iodine (VILI) has shown promising results compared to both Pap smear and VIA. In a multicenter study in several developing countries involving 56,939 women, the sensitivity of VIA varied from 67-79% and specificity ranged from 49%-86%, while the sensitivity and specificity of VILI varied from 76%–97% and 73%–92%, respectively [6]. VIA and VILI are inexpensive, require minimal infrastructure, allow for immediate treatment, and eliminate the need for a laboratory. Despite the advantages of VIA and VILI over cytology, these tests are subjective, requiring extensive and ongoing training of health-care providers, and are frequently associated with low specificity, which can result in significant overtreatment.

As an alternative to visual examination of the cervix, high resolution fiber optic microscopes are now available which facilitate direct visualization of neoplastic indicators such as elevated nuclear to cytoplasmic area ratio, nuclear crowding, and pleomorphic nuclei. These indicators are conventionally only observed during cytologic or histologic analysis following an invasive biopsy. Vital dyes can provide high optical contrast if applied topically. For example, proflavine, a fluorescent DNA label, distinguishes nuclei from the cytoplasm of the cell with nuclei appearing bright. Proflavine has peak absorbance at 445 nm and a peak emission at 510 nm. High-resolution fluorescence imaging of this agent in tissue can yield morphologic information such as the nuclear-to-cytoplasmic ratio which is an important parameter used in the histological diagnosis of cancer. It has a long history of safe clinical use as a topical antiseptic [8], [9], and is a component of acriflavine, which has been used in clinical imaging studies of the gastrointestinal tract and cervix [10], [11].

Due to their low cost and ease of interpretation at the point-of-care, high resolution optical imaging technologies may serve as an appropriate solution for cervical cancer screening in low resource settings [12]. We recently developed a high resolution microendoscope (HRME) to address the limitations of conventional methods of cervical cancer screening. The HRME has been evaluated in pilot studies of oral and esophageal precancer diagnosis [13]–[15]. In these studies, proflavine is applied topically to the squamous epithelium, and the area is interrogated with a fiber-optic bundle. Changes in cell morphology and epithelial architecture can be visualized in real time on a laptop computer.

The objective of this study was to evaluate the ability of this high-resolution microendoscope to identify cervical neoplasia in patients at the women's clinic at Princess Marina Hospital in Botswana. Images were acquired from 52 sites in 26 patients. The images acquired by the microendoscope were assessed by both visual inspection by two expert observers and quantitative analysis to discriminate neoplastic (≥CIN 2) from non-neoplastic cervical tissue. Results were compared to histopathology as the reference standard.

## Materials and Methods

### Instrumentation

**Figure 1:**
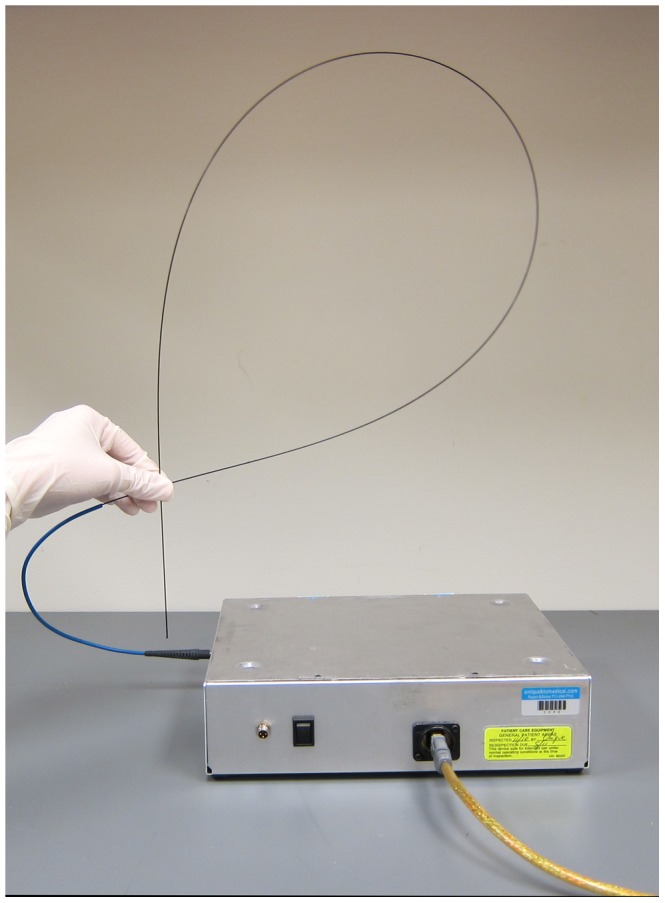
Photograph of the high resolution micro-endoscope (HRME).

Images were acquired using a custom designed, low cost high-resolution microendoscope (HRME); this system has been described in detail previously [16]. Briefly, the HRME is a fiber-optic fluorescence microscope which can acquire images of tissue with sub-cellular resolution at video-rate. As shown in **Fig. 1**, the HRME consists of a coherent, flexible 1 mm diameter fiber bundle (Sumitomo, IGN-08/30) coupled to a light source and a digital CCD camera. Light from a blue LED with peak wavelength centered at 455 nm (Thorlabs, FB450-40) provides illumination; fluorescence emission from the tissue is collected by the bundle, transmitted through a 475 nm dichroic mirror (Chroma, 475DCXRU), and focused onto an optical sensor of the CCD camera (Point Grey Research, GRAS-14S5C-M) by an objective lens (10×/0.25) and a 150 mm tube lens. Images are transferred to a laptop via IEEE-1394 (Firewire) cable. The field of view of the HRME is 720 µm in diameter, and the lateral spatial resolution is approximately 4 µm.

### Clinical Measurements

Women attending a colposcopy clinic on the basis of an abnormal Pap smear were eligible to participate in the study if they were at least 18 years of age and not pregnant. A health care provider described the procedure to eligible patients; patients who agreed to participate were provided with and signed forms indicating informed consent and authorization.

Study participation included colposcopic examination of the vulva, vagina, and cervix using 3% acetic acid. Abnormal cervical lesions in the transformation zone were identified and noted by the study physician, in accordance with routine patient care. Each lesion was described and classified by the study physician as clinically high grade, clinically low grade, or clinically normal using the Reid Colposcopic Index [17]. Following routine colposcopic examination, a solution of proflavine hemisulfate (0.01% w/v in sterile phosphate buffered saline) was topically applied to the cervix with a cotton swab. Following application of proflavine, the HRME was advanced through the speculum and placed in gentle contact with the cervix. Three second movies were obtained by the HRME from up to two clinically abnormal cervical sites and one clinically normal cervical site in the transformation zone. Following optical measurements, all sites interrogated with the HRME probe were biopsied and submitted for routine histopathologic analysis. Biopsies were read by a single pathologist who was blinded to the results of the optical imaging. Diagnostic classification categories included normal, inflammation, HPV effect, grade 1 cervical intraepithelial neoplasia (CIN 1), grade 2 cervical intraepithelial neoplasia (CIN 2), and grade 3 cervical intraepithelial neoplasia (CIN 3) using standard histopathologic criteria [18]. Squamous metaplasia was considered a normal result. No cases of *carcinoma in situ* (CIS) or squamous cell carcinoma (SCC) were observed in this study. Low grade dysplasia includes HPV effect and CIN 1, and high grade dysplasia includes CIN 2 and CIN 3. Normal/benign results included inflammation. For purposes of data analysis, neoplastic tissue was classified as high grade dysplasia, and all other diagnoses were considered to be non-neoplastic.

### Data Analysis

For each HRME movie, one representative frame was chosen which minimalized motion artifact and obstruction of the field of view due to cellular debris. The user selecting the frames was blinded to all diagnostic information. The resultant HRME images were reviewed for quality control by a reviewer blinded to all diagnostic information. Images were discarded if more than 50% of the field of view was out of focus, contained evidence of motion artifact, or contained significant debris. HRME images which passed quality control and had a corresponding histopathologic diagnosis were analyzed in two ways.

First, images were reviewed by two expert observers, who had each previously reviewed HRME images from more than 50 cases of normal and neoplastic tissues. HRME images passing quality control were presented to each reviewer in randomized order; reviewers were instructed to classify each image as non-neoplastic or neoplastic. The sensitivity and specificity of qualitative analysis was calculated for each reviewer using histopathologic diagnosis as the reference standard.

Second, image analysis software was used to analyze each HRME image which passed quality control to calculate quantitative image features. Analysis focused on assessment of nuclear size, since changes in nuclear size and nuclear-to-cytoplasmic ratio are hallmark histopathologic features of cervical precancer [19]. A reviewer, blinded to the histopathologic diagnosis, first manually selected a region of interest (ROI) for quantitative analysis. The ROI was selected to include regions with visible nuclei and to exclude regions with evidence of motion artifact, image saturation, or debris. Following selection of the ROI from an HRME image, image contrast was adjusted using a contrast-limited adaptive histogram equalization, and median filtering was applied to remove the background pattern associated with the structure of the fiber bundle. Each processed gray-scale image was then converted to a binary image using a threshold value. A reviewer, again blinded to the histopathologic diagnosis, reviewed the binary images and adjusted the threshold value to ensure that nuclei were segmented appropriately based on visual assessment. Binary images were then processed to remove all objects with fewer than 50 pixels (assumed to be noise) and more than 1500 pixels (assumed to be debris). Finally, for each image the mean nuclear to cytoplasmic area (N/C) ratio was calculated by dividing the total nuclear area by the total cytoplasmic area within the ROI.

### Ethics Statement

The study protocol was reviewed and approved by the Institutional Review Board at Rice University and the Health Research Division Office of the Botswana Ministry of Health. Written informed consent documents were available in both English and the local national language (Setswana). All patients involved in the study gave written informed consent.

## Results

HRME images were acquired from 52 sites in 26 patients. Images from 8 sites were excluded from further analysis because they did not pass quality control criteria outlined above. The rejection of the 8 sites eliminated all data from one subject. For this subject, the two movies collected were both out of focus because the distal tip of the probe was not in contact with the cervical epithelium. **Table 1** summarizes the histopathologic diagnoses of the remaining 44 sites. The majority of patients in the study were human immunodeficiency virus (HIV) infected, which has been correlated with a high incidence of intraepithelial lesions [20].

**Table 1:** Number of sites imaged by HRME, grouped according to histopathology diagnosis.

Diagnostic Category	Histopathology Diagnosis	Number of Sites Imaged
Normal/Benign	Normal	5
	Inflammation	7
Low Grade Dysplasia	HPV Effect	11
	CIN I	7
High Grade Dysplasia	CIN 2	2
	CIN 3	12
**Total Number of Sites Imaged**		**44**

High grade dysplasia was considered neoplastic.

All other categories were considered non-neoplastic.

**Figure 2:**
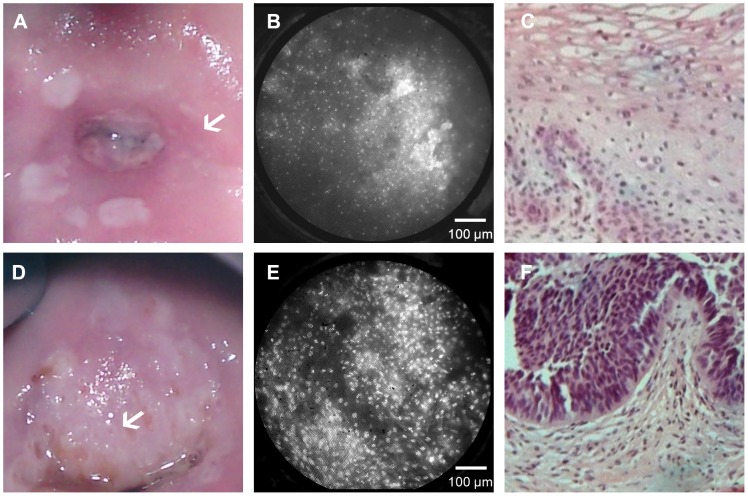
Comparison of colposcopic appearance, HRME images, and histologic diagnosis. The top row shows images obtained from a clinically normal region of the cervix. The white arrow in the colposcopic image (a) indicates the area imaged with the HRME. The HRME image (b) shows small, uniformly spaced nuclei, and was considered non-neoplastic by both subjective expert observers, which is consistent with histopathology indicating HPV effect (c). The bottom row shows images from a clinically abnormal region of the cervix. The white arrow in the colposcopic image (d) indicates a region with a clinical impression of high grade disease. The corresponding HRME image (e) shows large, pleomorphic, crowded nuclei and was considered neoplastic by both subjective expert observers, which is consistent with histopathology indicating CIN3 (f).

**Figure 2** shows typical HRME images of clinically and histopathologically normal **(a–c)** and neoplastic **(d–f)** cervical tissues. The top row shows a colposcopic photograph of the cervix; the white arrow indicates the clinically normal site where the HRME probe was placed. The corresponding HRME image shows small, regularly spaced nuclei. This image was considered qualitatively non-neoplastic by both expert reviewers, which was consistent with the histopathologic diagnosis of HPV effect. The bottom row shows a colposcopic photograph indicating a clinically abnormal thick aceto-white lesion (white arrow). The HRME image obtained from this site shows large, crowded, pleomorphic nuclei. The image was considered qualitatively neoplastic by both expert observers, which was consistent with the histopathologic diagnosis of CIN 3.

**Figure 3:**
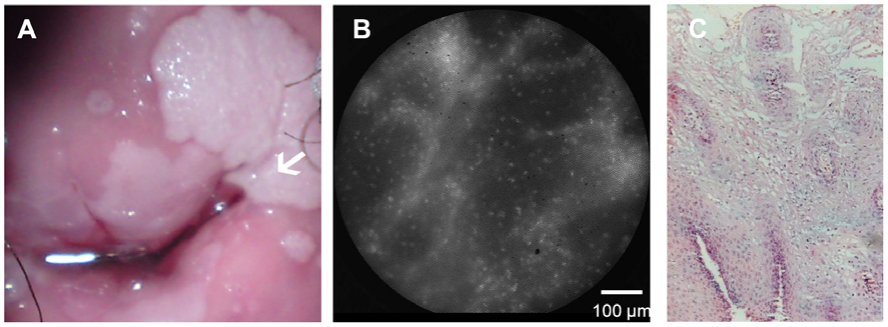
Comparison of colposcopic appearance, HRME images, and histologic diagnosis in a case where colposcopic impression did not agree with histopathologic diagnosis. The white arrow in the colposcopic image (a) indicates the area imaged with the HRME; the clinical impression of this warty looking lesion was high grade disease according to the Reid Colposcopic Index. The HRME image (b) shows small, uniformly spaced nuclei. This image was considered qualitatively non-neoplastic by both subjective expert observers, which is consistent with the histologic image indicating HPV effect (c).

**Figure 3** shows HRME images obtained from a case in which the colposcopic impression did not agree with the histopathologic diagnosis. The colposcopic photograph shows a warty lesion (white arrow) which was classified as clinically high grade disease according to the Reid Colposcopic Index. The HRME image obtained from this site showed small nuclei, characteristic of normal epithelium. The expert reviewers categorized this image as non-neoplastic. This was consistent with the histopathologic diagnosis of HPV effect.

Qualitative diagnoses by the expert HRME observers for all 44 sites were compared with histopathologic diagnosis to calculate sensitivity and specificity. Observer #1 classified the images with a sensitivity and specificity of 86% and 70% respectively. Observer #2 classified the images with a sensitivity and specificity of 93% and 73% respectively.

**Figure 4:**
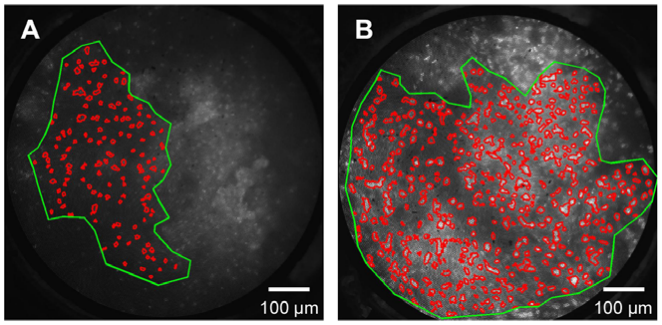
HRME images were analyzed to calculate the average nuclear to cytoplasmic (N/C) area ratio. A region of interest (green) was defined by the user to select regions of the image with visually observable nuclei. Within this region, a computer program segmented nuclei and calculated the N/C area ratio. Representative images showing a site diagnosed as HPV with an N/C ratio of 0.08 (a) and site diagnosed as CIN 3 with an N/C ratio of 0.22 (b) are shown.

Quantitative morphometric image analysis was next performed; **Figure 4** illustrates results from representative HRME images of non-neoplastic and neoplastic tissues. In each image, the user-defined ROI is outlined in green; within this ROI, the automatically segmented nuclei are outlined in red. ROI size did not vary significantly from neoplastic images to non-neoplastic images (Student's t-test, p>0.5). **Figure 4a** shows an HRME image from a site histopathologically diagnosed as HPV effect; the N/C ratio was 0.08. In contrast, **Fig. 4b** shows an HRME image from a site diagnosed as CIN 3 with an N/C ratio of 0.22.

**Figure 5:**
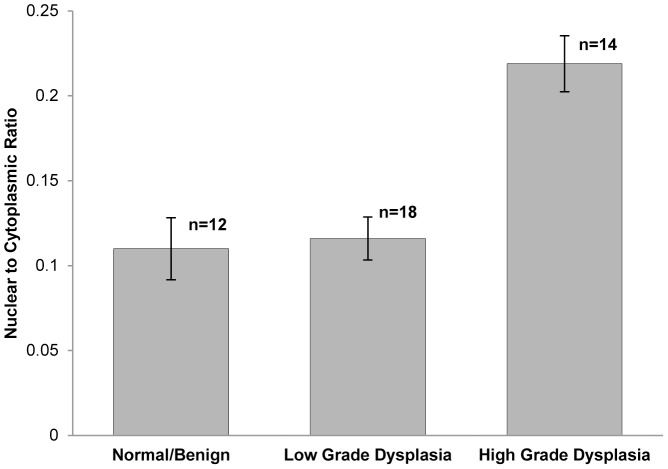
The average N/C ratio versus histopathologic diagnosis by diagnostic category. The average N/C ratio of sites diagnosed as high grade dysplasia was significantly higher than those diagnosed as normal/benign (Student's t-test, p = 2.0E-5) and low grade dysplasia (Student's t-test, p = 1.9E-5). Error bars indicate standard error.

To assess whether differences in the N/C ratio as measured from HRME images correlated with histopathologic diagnosis, we grouped sites by histopathologic diagnosis and calculated the mean of the N/C ratio (**Fig. 5**). Results show that the average N/C ratio increases with increasing grade of neoplasia; the mean N/C ratio of sites diagnosed as high grade dysplasia was significantly higher than those diagnosed as normal/benign (Student's t-test, p = 2.0×10^−5^) and those diagnosed as low grade (Student's t-test, p = 1.9×10^−5^).

**Figure 6:**
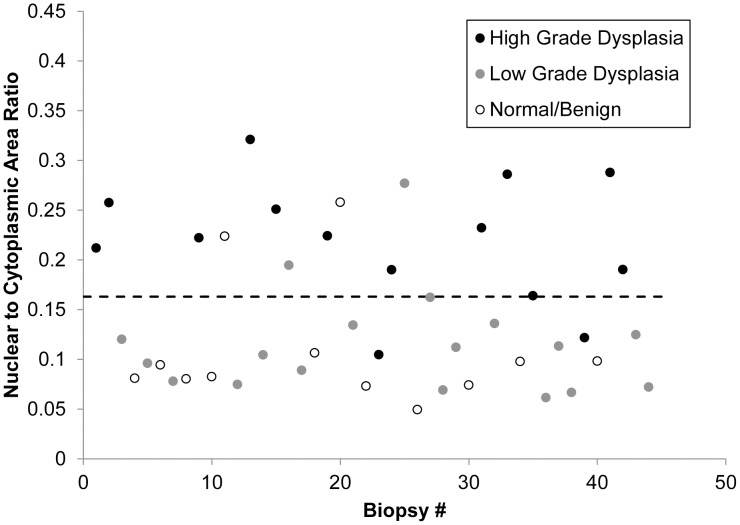
Scatter plot of N/C ratio for all samples, broken down by histopathologic diagnosis. The dotted line indicates a threshold at 0.163 with a sensitivity of 86% and a specificity of 87% for separating samples diagnosed as high grade dysplasia from samples diagnosed as low grade dysplasia or normal/benign.

**Figure 6** shows a scatter plot indicating the N/C ratio vs. histopathologic diagnosis for each of the 44 sites measured in this study. We explored whether this parameter could be used to classify sites as neoplastic (high grade dysplasia) or non-neoplastic (normal/benign or low grade dysplasia). Using a simple cut-off where sites with an N/C ratio greater than 0.163 were diagnosed as neoplastic and sites with an N/C ratio of less than 0.163 were classified as non-neoplastic correctly classified 12 of 14 sites with a histopathologic diagnosis of high grade, 16 of 18 sites with a histopathologic diagnosis of low grade, and 10 of 12 sites with a histopathologic diagnosis of normal/benign.

**Figure 7:**
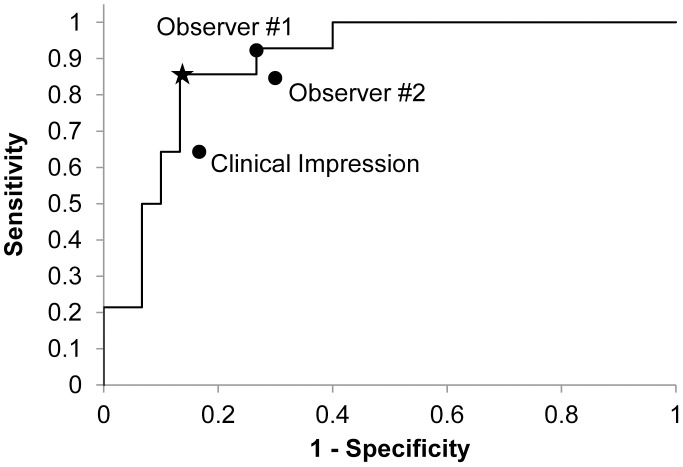
Receiver operator characteristic curve for the algorithm shown in **Figure 6**. The star indicates the Q-point, with a sensitivity of 86% and a specificity of 87%. The performance of visual interpretation of HRME images by two observers is indicated and falls along the ROC curve for automated image analysis. For comparison, the sensitivity and specificity of clinical colposcopic impression for the same sites were 64% and 83%, respectively.

The receiver operator characteristic (ROC) curve for a diagnostic algorithm based on the N/C ratio is shown in **Fig. 7**. The Q-point indicated on the figure corresponds to the cut-off value shown in **Fig. 6**, and corresponds to a sensitivity of 86% and a specificity of 87%. **Figure 7** also shows the accuracy of visual interpretation of the HRME images by two observers relative to the reference standard of histopathology. For both observers, the accuracy of visual image interpretation lies near the ROC curve for quantitative image interpretation. For comparison, the sensitivity and specificity of clinical colposcopic impression for the same sites were 64% and 83%, respectively when clinically low grade lesions were considered non-neoplastic.

## Discussion

This study suggests that high resolution microendoscopy shows promise to improve cervical cancer screening in resource-constrained settings. In this pilot study, both the sensitivity and specificity of HRME based detection were higher than that of colposcopic impression.

While encouraging, our study has a number of limitations. First, it was a pilot study involving only 52 sites in 26 patients. A larger, prospective study is required to confirm the results. Second, all HRME images were acquired from colposcopically suspicious and normal areas in the transformation zone. We have not characterized HRME images of endocervical tissue to determine whether they may be a potential source of error. Although all data in this study was taken from the transformation zone of the cervix as identified by the physician, three biopsies were noted to contain predominantly columnar epithelium by pathology. Further research is necessary to identify the differences between HRME images of dysplastic squamous epithelium, normal columnar epithelium, and dysplastic columnar epithelium.

High resolution imaging offers a number of potential advantages as a screening tool. Studies of VIA have shown a wide range of resulting sensitivities and specificities. A metastudy of VIA, which included Sankaranarayanan's multicenter study, revealed sensitivities ranging from 41%–92% and specificities ranging from 49%–98% [21]. This significant variation in accuracy calls for additional measures to maintain consistency of diagnoses. The HRME system could potentially act as additional test to VIA and VILI screening systems.

When VIA and VILI screening systems refer patients for additional treatment, providers in low resource settings can choose to screen and provide care in one visit. The “see and treat” system eliminates biopsies to confirm disease in visible lesions before a loop electrosurgical excision procedure (LEEP) or cautery [22]. Our results suggest that the HRME shows promise as a reliable alternative to a biopsy at this second line of care in resource-constrained areas. In particular, the high specificity of the HRME results indicates that high resolution microendoscopy could potentially lower the number of overtreated lesions.
